# Development, Characterization, and In Vitro Evaluation of Tamoxifen Microemulsions

**DOI:** 10.1155/2012/236713

**Published:** 2012-01-05

**Authors:** E. Monteagudo, Y. Gándola, L. González, C. Bregni, A. M. Carlucci

**Affiliations:** ^1^Department of Pharmaceutical Technology, Faculty of Pharmacy and Biochemistry, University of Buenos Aires, Junín 956, 1113 Buenos Aires, Argentina; ^2^Institute of Biochemistry and Biophysics, (IQUIFIB), National Science Research Council (CONICET), Junín 956, 1113 Buenos Aires, Argentina

## Abstract

Microemulsions (MEs) were designed by an innovative rational development, characterized, and used to load up to 20 mM of Tamoxifen citrate (TMX). They were made with acceptable and well-characterized excipients for all the routes of administration. Some of their properties, such as nanometric mean size and long stability shelf life, make them interesting drug delivery systems. The results obtained after the in vitro inhibition of estradiol-induced proliferation in MCF-7 breast cancer cells demonstrated a significant effect in cell growth. A decreasing of at least 90% in viable cells was shown after the incubation with MEs containing 20 mM of TMX. Besides, two compositions which loaded 10 mM of drug showed a cytotoxic effect higher than 70%. These results encourage the evaluation of alternative protocols for this drug administration, not only for estrogen receptor (ER) positive tumors, but also for ER negative.

## 1. Introduction

One of the major problems facing cancer therapy is administering the required therapeutic concentration of the drug at the tumor site for the desired period of time. Targeted drug delivery to solid tumors is necessary in order to achieve optimum therapeutic outcomes. It would, therefore, be desirable to develop chemotherapeutics that can either passively or actively target cancerous cells. Passive targeting exploits the characteristic features of tumor biology that allow nanocarriers to accumulate in the tumor by the enhanced permeability and retention (EPR) effect [1]. Whereas free drugs may diffuse nonspecifically, a nanocarrier can extravasate into the tumor tissues via the leaky vessels by the EPR effect. The dysfunctional lymphatic drainage in tumors retains the accumulated nanocarriers. Particles with diameter <200 nm resulted in the most effective ones [2, 3].

Microemulsions (MEs) are extensively studied nanocarriers; they are defined as a system of water, oil, and amphiphile which is a single optically isotropic and thermodynamically stable liquid solution. Their structure consists in micro-domains of lipids or water stabilized by an interfacial film of surfactant and cosurfactant molecules. They can be classified as oil in water (o/w) or water in oil (w/o) and the droplet size is lower than 150 nanometers. They present a number of advantages as drug delivery system, such as the ability to solubilize hydrophobic drugs, spontaneous assemble, long-term physical stability, and ease of manufacturing [4]. They presented successful results for all administration routes. There have also been of an increasing interest for their administration via the parenteral route [5, 6], due to the number of acceptable excipients available nowadays [7, 8].

**Figure 1:**
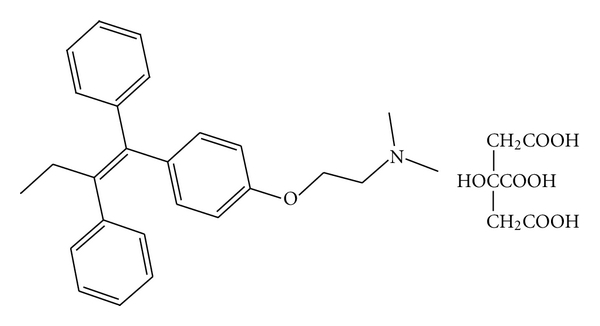
Chemical structure of tamoxifen citrate.

Tamoxifen citrate (TMX) (Figure 1), is an antiestrogen, nonsteroidal derivative of triphenylethylene with poor water solubility [9], that is widely used in hormone therapy and breast cancer prevention even in an advanced stage. Its use is especially indicated for postmenopausal women who have estrogen-receptor- (ER-) positive breast cancer. It is an estradiol competitive inhibitor for the estrogen receptor. It inhibits proliferation by arresting the cell cycle and induces breast cancer cells apoptosis [6, 10, 11]. It is also thought to induce a tumoricidal effect on estrogen receptor-negative cells by increasing the secretion of inhibitory growth factors. Recent reports have shown that TMX may possess antiangiogenic activity through its antiestrogenic effects [1].

 TMX is administered by oral route in dose ranges from 20 to 40 mg a day, but up to 200 mg a day has been reported [12]. Regarding pharmacokinetics, its oral bioavailability is affected by the first pass effect and is a substrate for some protein families that mediate toxic compounds efflux outside the organism [13]; it also presents vulnerability to enzymatic degradation in both intestine and liver. Following long-term therapy, TMX has some major side effects, including higher incidence of endometrial cancer, liver cancer, thromboembolic disorders, and development of drug resistance [1].

To address the challenges of targeting tumors with nanotechnology, it is necessary to combine the rational design of nanocarriers with the fundamental understanding of tumor biology. It is to remark that an increasing number of nanovectors are currently being tested for breast cancer treatment, including liposomes and albumin-bound paclitaxel as examples [14].

Because of the above-mentioned reasons, TMX represents a promising lipophilic model drug either for oral or parenteral administration using MEs as passive targeting drug delivery system. Therefore, an alternative protocol for oral, IM, or IV administration in breast cancer or in ER-negative tumors would be evaluated taking advantage of ME properties [15].

The aim of the present work was to design and characterize o/w MEs composed by pharmaceutically accepted excipients for TMX delivery. They would be further proposed for alternative protocols of oral or parenteral administration. The biological behavior of the selected compositions for passive targeting drug delivery was also evaluated in MCF-7 human breast cancer cell line. 

## 2. Materials and Methods

### 2.1. Material

Phosphatidylcholine (PC, Phospholipon 90 NG) was purchased from Phospholipid, Germany; Polyoxyethylene Sorbitan Monooleate (Polysorbate 80, PS 80) was from Fisher Chemicals, NJ, USA; Tamoxifen citrate was from Saporiti S.A., Buenos Aires, Argentina; ethanol was bought at J. T. Baker, USA; Capmul MCM L (glycerol monocaprylocaprate) and Captex 355 (caprylic/capric Triglyceride) were purchased from Abitec, Columbus, USA. Estradiol was from Sigma Aldrich. St. Louis, MO, USA. Imwitor 408 (propylene glycol caprylate) and Myiglyol 840 (propylene glycol dicaprylate/dicaprate) were from Sasol, Witten, Germany. Oleic acid and Isopropyl mirystate were from Merck, Germany. Propylene-glycol and polyethylene glycol 400 were bought at BASF, NJ, USA. Labrafil M 1944 CS (oleoyl macrogolglycerides (polyoxylglycerides) and Transcutol P (diethylene glycol monoethyl ether) were purchased from Gatefossé, France. All reagents were of analytical grade. Distilled water was obtained from a Milli-Q equipment.

### 2.2. Preliminary Solubility Evaluation for the Screening of Components

PS 80 was selected as surfactant model because it is listed as a generally recognized as safe (GRAS) excipient. In addition, it is extensively used for different ways of administration, including the parenteral route [16], and for microemulsions' preparation [8].

The solubility of TMX in a number of excipients was estimated. They were Isopropyl myristate (IPM), Mygliol 840, Captex 355, Oleic acid, Imwitor 408, phosphatidylcholine (PC) and Capmul MCM L. PC is solid at room temperature, so a suspension was prepared (being 16% m/v the maximum concentration tested). These oils are widely used as no polar phases for ME formulation [17, 18]. PC has also been used for the formulation of parenteral MEs [19].

Regarding cosurfactants, five compounds were tested: Ethanol, Polyethilenglycol 400 (PEG 400), Transcutol P, Labrafil 1944 CS, and Propylenglycol (PG). All of them are included in the FDA inactive ingredients guide.

To determine the drug solubility of TMX in excipients, drug in excess was added until turbidity was reached. Then, the samples were left to equilibrate using a Rotating Bottle apparatus (Varian, USA) at 5 RPM. If the solution was clear after rotation for a short time, a more active compound was added. Otherwise, the sample was left to equilibrate for 72 hours and it was, then, filtered using 0.45 *μ*m PVDF membranes (Pall life sciences, USA). The filtered sample was analyzed by HPLC.

Quantitative determinations of TMX were performed using a Shimadzu Class VP HPLC. The chromatographic conditions were: column Zorbax Eclipse XDB Phenyl with detection at 254 nm; temperature was fixed at 35°C. The mobile phase was constituted by methanol (1000 mL), water (320 mL), acetic acid glacial (2 mL), octansulphonate (1.08 g), and triethylamine (1 mL).

These same conditions were also used for the determination of solubilizing capacity shown by formulations. All experiments with TMX were carried out using amber glass material due to drug photosensitivity.

### 2.3. Preliminary Cytotoxicity Assay

Although nonionic surfactants are considered less toxic than ionic surfactants, they are often reported as responsible for a number of adverse effects [20]. This is the main issue that pharmaceutical design has to overcome when formulating MEs, because high levels of surfactants are sometimes needed.

To assess the extent in which PS 80 could affect cell viability, a cytotoxicity assay using different concentrations was performed (5, 10, 20, and 25% m/v). The five co-surfactants in solutions of 35% m/v and the seven lipids in suspensions of 4% and 16% m/v were also evaluated.

For cytotoxicity studies, cells were seeded in clear 96-well plates (Corning Costar, Fisher Scientific, USA) at a density of 10,000 cells/well. After 24 hours, 5 *μ*L of the samples were added in 200 *μ*L of medium. Cells were incubated at 37°C for 48 hours in a 5% CO_2_ atmosphere. Finally, the amount of viable cells was determined using CellTiter 96 AQueous Nonradioactive Cell Proliferation Assay (MTS), Promega.

### 2.4. Pseudoternary Phase Diagram Construction

Based on solubility and preliminary cytotoxicity results, excipients were selected to perform ME region screening. Different amounts of PS 80 and each one of the selected co-surfactants and oil phases were mixed using magnetic stirrer during 10 minutes. Then, water was added and samples were left to equilibrate using a thermal bath at 37°C (Varian, USA) for 1 hour. The adopted criteria used for considering a formulation as an ME was based on the visual analysis of the compositions searching for clear, single-phase, isotropic and low-viscous systems.

### 2.5. Screening and Optimization of MEs

Once the screening was finished, a number of compositions were selected on basis of noncytotoxic effect of their components and also on a high TMX solubilizing capacity. After that they were evaluated for MCF-7 cells' survival as described above.

### 2.6. Preparation of TMX-Loaded MEs

TMX-loaded MEs were prepared by weighing appropriate amounts of PS 80, the co-surfactant, and oil phase selected according with previous adopted criteria; gentle magnetic stirring during 10 minutes at room temperature was applied so as to obtain homogenous samples, which were left to equilibrate using a thermal bath at 37°C (Varian, USA) for 1 hour. Next, three different amounts of TMX were added and dissolved with magnetic stirring. Finally, the corresponding amount of water for each one of the selected compositions was added under agitation at room temperature. 

### 2.7. Physicochemical Characterization of TMX-Loaded MEs

Density was measured using a Mettler Toledo 30 px. Formulation of pH was determined with a pHmeter Mettler Toledo seven easy. Conductivity was assessed using an Accumet research AR20 at 25°C; for rheological measurements a Brookfield DV-III Ultra at 25°C was used. Polarization microscopy was performed using an Olympus BH microscope [21].

Droplet size was analyzed with a Nanozetasizer ZS, Malvern Instruments, UK. Samples were not diluted to carry out the measurements and assays were performed at 25°C. The polydispersity index indicates the size distribution within a ME population. The *z* potential of the formulations was determined using the same equipment (Nanozetasizer ZS, Malvern Instruments, UK). Samples of the formulation were placed in the electrophoretic cell, where an electric field of about 15 V/cm was applied. The electrophoretic mobility measured was converted into *z* potential using the Smoluchowski equation.

The morphology of MEs was studied using transmission electron microscopy (TEM). The MEs were first diluted in water (1 : 40), a sample drop was placed onto a grid covered with Formvar film and the excess was drawn off with a filter paper. Samples were subsequently stained with uranyl acetate solution for 30 s. Samples were finally dried in a closed container with silica gel and analyzed. The droplet diameter was estimated using a calibrated scale.

Chemical stability was performed using the HPLC equipment described for solubility assays (Shimadzu Class VP HPLC), and the chromatographic conditions were also the same. For short time stability studies, samples were left on the bench at room temperature for a month and, then, were reanalyzed. Direct observation of the formulations was used to evaluate drug precipitation or other physical change during the evaluation period.

The objective of thermodynamic stability is to evaluate the phase separation and effect of temperature variation on MEs formulation. All the MEs prepared were centrifuged (Eppendorf Centrifuge 5810) at 15,000 rpm for 15 min, and then they were observed visually for phase separation. Formulations that did not show any sign of phase separation after centrifugation were subjected to freeze thaw cycle. In a freeze thaw study, TMX MEs were evaluated for two freeze thaw cycles between (−20°C and +25°C) with storage at each temperature for not less than 4 h [22].

### 2.8. Cell Culture Conditions

MCF-7 human breast cancer cell line was obtained from the American Type Culture Collection (ATCC) (Rockville, MD, USA). Cells were maintained in Dulbecco's minimum essential medium (DMEM) supplemented with 10% fetal bovine serum (FBS), 50 *μ*g/mL gentamycine (Invitrogen Argentina), and 2 mM L-glutamine (Invitrogen Argentina). Cells were cultured in 75 cm^2^ culture flasks at 37°C in a humidified atmosphere of 5% CO_2_.

### 2.9. Cytotoxicity and In Vitro Performance of the Selected TMX-Loaded MEs

For in vitro performance studies, cells were seeded in 96-well plates at a density of 5,000 cells/well. After 24 hours, medium was replaced by phenol-red-free media containing 2 mM L-glutamine for 24 hours. To analyze effects of selected TMX-loaded formulations, cells were subsequently incubated with estradiol 10 nM and the TMX-loaded MEs; in parallel, a TMX suspension containing 10 mM of drug in presence and in absence of estradiol was also evaluated. Cells were incubated further for 48 hours and then cell viability was assessed by the cell proliferation assay (MTS).

### 2.10. Statistical Analysis

Statistical calculations were performed with the GraphPad InStat statistical package for Windows. Data shown in tables and figures of in vitro properties evaluation represent mean of three determinations ± standard deviation (SD). Statistical significance of the differences between the groups was calculated by the Tukey-Kramer multiple comparison test and probability value of *P* smaller than 0.05 indicated a statistically significant difference.

## 3. Results and Discussion

### 3.1. Preliminary Solubility Evaluation

**Figure 2:**
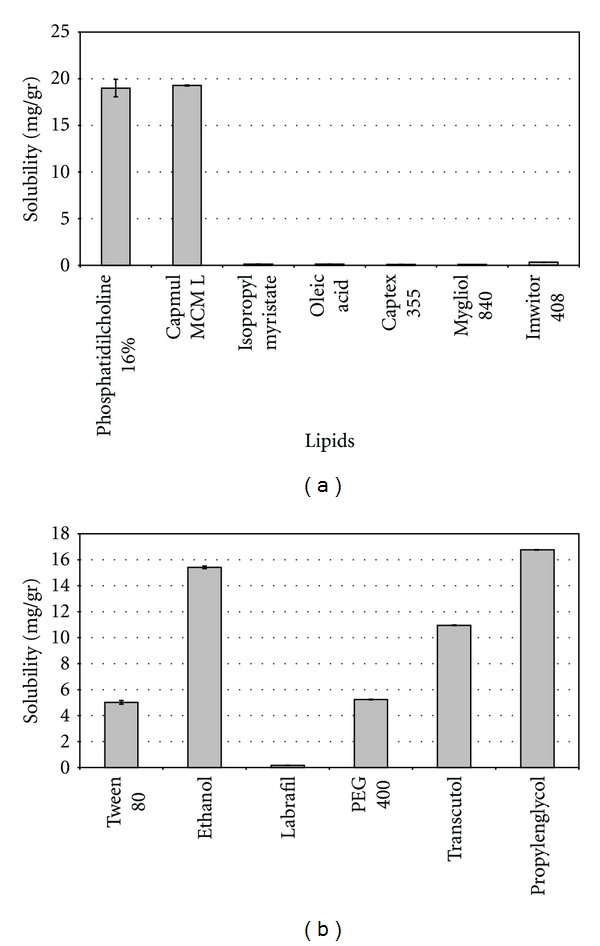
(a) Solubility of Tamoxifen citrate in oil phases (expressed in mg/g). Each bar represents the mean of three samples ± SD. (b) Solubility of Tamoxifen Citrate in Polysorbate 80 and cosurfactants (expressed in mg/g). Each bar represents the mean of three samples ± SD.

TMX resulted almost insoluble in IPM, Mygliol 840, Captex 355, Oleic acid, and Imwitor 408 and showed solubility near 20 mg/g in the PC suspension and in Capmul MCM L (Figure 2(a)). Therefore, only PC and Capmul MCM L were selected for the forthcoming screening. The selection of the oily phase is very important because the drug solubility in the formulation depends mainly on it [23, 24]. So, this property results, fundamental in the search for high solubilizing capacity systems.

 Lipid solubility values found in this work are in accordance with previous studies and significantly higher compared to other lipids not considered in this study [25]. They also were significantly higher than TMX solubility in water (*≈*20 mg/mL and *≈*0.4 *μ*g/mL, resp.). Furthermore, the high solubility in PC is in accordance with previous works [26], which stated that active compounds with an intermediate lipophilicity (Log *P* of 4.0 and above, being 7.9 the value of the Tamoxifen) have a high tendency to be solubilized by phospholipids.

Solubility of TMX in the five co-surfactants and in PS 80 is depicted in Figure 2(b). The highest solubilizing capacity was achieved with PG and ethanol; therefore, both compounds were selected to act as coemulsifiers in the forthcoming ME screening. However, TMX showed a considerable solubility in PEG 400 and Transcutol *P*, but it resulted significantly lower than the selected compounds (*P* < 0.05). Finally, Labrafil 1944 CS was discarded because it was the co-surfactant with the lowest drug solubilizing capacity.

Solubility of TMX in PS 80 was around 5 mg/g; however, it is expected that these results slightly impact on the final therapeutic agent solubilization. The most important factor that contributes to the final ME solubilizing capacity in poorly water soluble drugs is the solubility in the lipid internal phase [26].

### 3.2. Preliminary Cytotoxicity Study

In order to avoid interference when testing selected vehicles for in vitro performance, a preliminary cytotoxicity experiment on the MCF-7 cancer cell line was performed.

**Figure 3:**
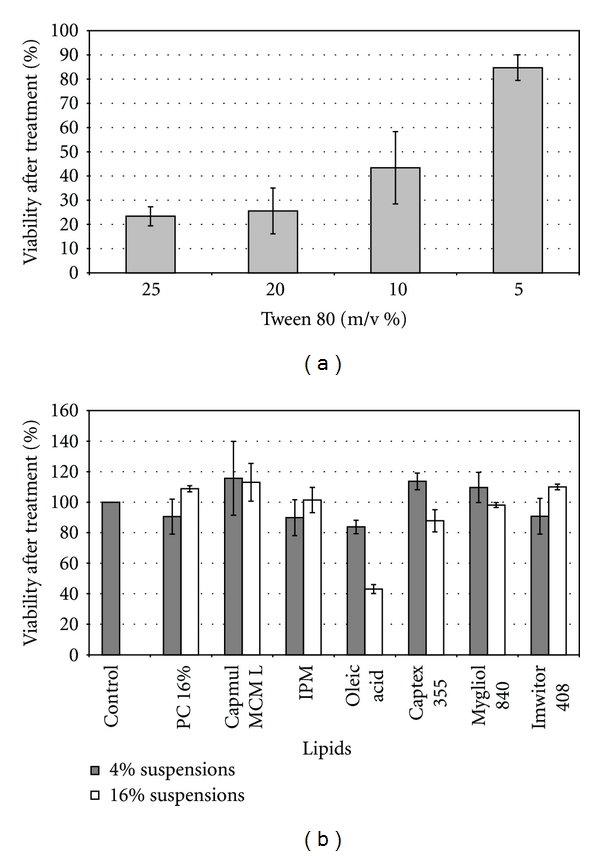
(a) Cell viability of MCF-7 breast cancer cells incubated at 37°C for 48 hrs with Polysorbate 80 at 25, 20, 10, and 5% m/v, respectively. Each bar represents the mean of three samples ± SD. (b) Cell viability of MCF-7 breast cancer cells incubated at 37°C for 48 hrs with suspensions of 4% and 16% of each one of the selected lipids. Each bar represents the mean of three samples ± SD.

As it can be observed in Figure 3(a), only samples containing 5% m/v of PS 80 exhibited low cytotoxicity; higher concentrations than 5% m/v showed a percentage of cell viability after treatment lower than 50%. Therefore, it can be concluded that formulations containing PS 80 at concentrations above 5% would be toxic to the cells. Because of it, false-positive results could be addressed when evaluating their in vitro performance. As a result of the preliminary surfactant cytotoxicity experiments and in order to avoid excipient related effects on the cells, final formulations have been diluted prior to their in vitro performance evaluation. Oleic acid was the only no polar phase associated with cytotoxicity effect at both assayed concentrations (Figure 3(b)). Labrafil CS was the only cosurfactant which showed that inconvenience.

### 3.3. Screening and Optimization of MEs

Based on solubility and cytotoxicity results, the following excipients were selected to perform the preliminary microemulsion screening: PS 80 as surfactant, ethanol, and PG as co-surfactants and PC and Capmul MCM L as the oil phases.

Once the screening was finished, a number of compositions which resulted to be isotropic were selected and are shown in Table 1. The selection included compositions with a relative proportion of PS 80 lower than 20%, relative concentrations of each one of the oil phases between 8 and 16%; the level of the co-surfactants was fixed in 25%. None of these compositions containing PG as cosurfactant, matched the adopted criterion for considering ME system and they were discarded for the next step of selection.

**Figure 4:**
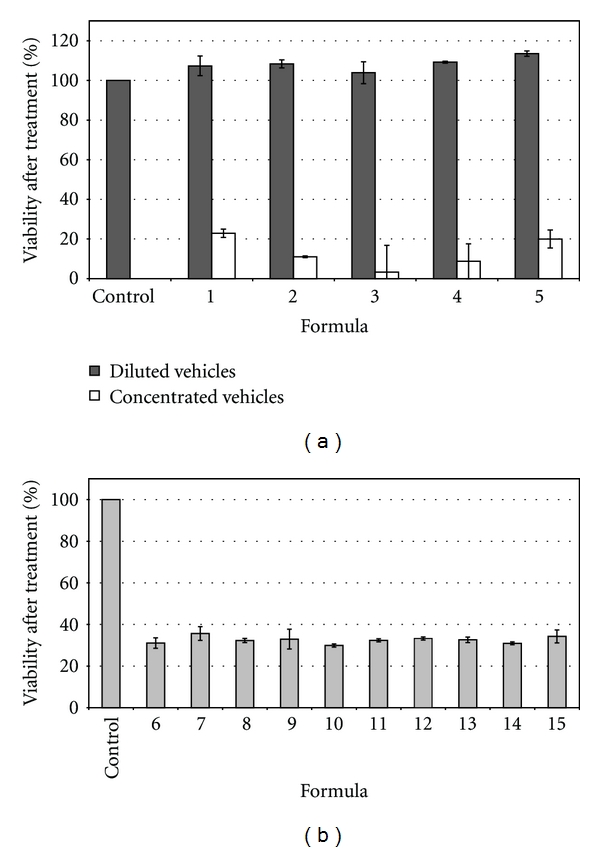
(a) Cell viability of MCF-7 breast cancer cells incubated at 37°C for 48 hrs with selected microemulsions N° 1 to N° 5, after a dilution (1 : 5) and without dilution. Each bar represents the mean of three samples ± SD. (b) Cell viability of MCF-7 breast cancer cells incubated at 37°C for 48 hrs with selected microemulsions N° 6 to N° 15 after 1 : 5 dilution. Each bar represents the mean of three samples ± SD.

In relation to Capmul MCM L, promising results were observed in agreement with other authors; as it has been recorded medium chain monoglycerides are known for their ease of emulsification when compared to fixed oils or long-chain fatty acids [5, 18]. They also exhibit good solubilizing capacity. However, this oil phase could not be forthcoming evaluated in MEs' selection because of the high cytotoxicity exhibited in cell cultures. Formulations containing Capmul MCM L as oil phase were highly cytotoxic even though they were diluted to avoid surfactant toxicity and that the lipid alone did not show that property (Figures 4(a) and 4(b)). In this case, cytotoxicity may be due to the effect of the lipid on cells when delivered by ME. For this reason, MEs containing Capmul MCM L were discarded for the in vitro inhibition of proliferation experiments and their pseudoternary phase diagrams are not shown.

**Figure 5:**
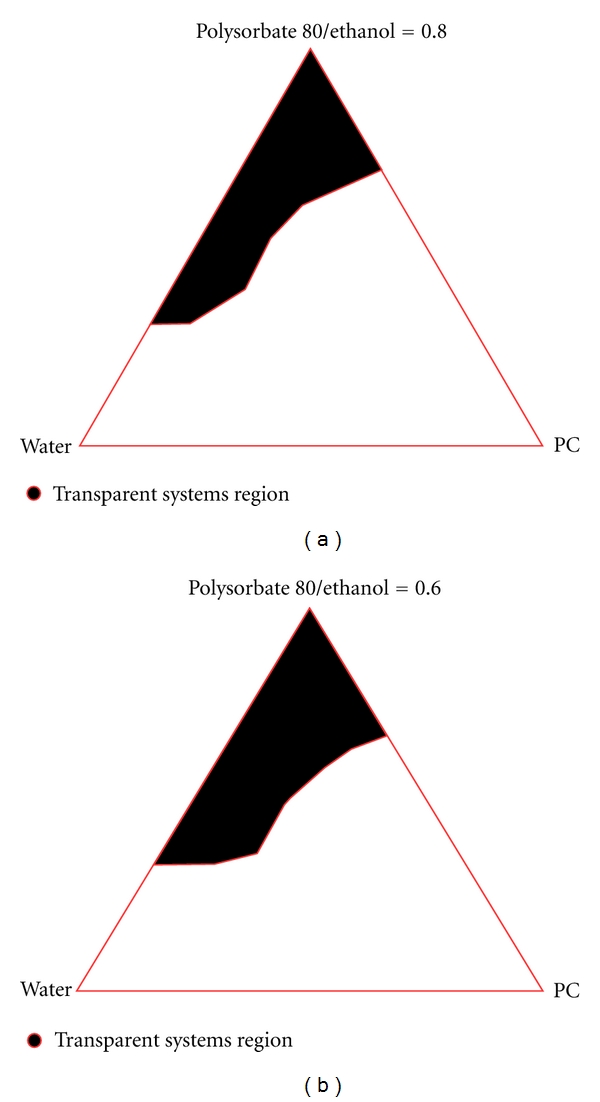
Pseudoternary phase diagrams of the selected formulations (Ratios Polysorbate 80: ethanol 0.8 and 0.6, resp.).

At this stage of the work, only MEs containing PC, ethanol, and PS 80 were selected. For their pseudoternary phase diagrams construction, two different surfactant/co surfactant ratios: 0.8 and 0.6 were considered (Figures 5(a) and 5(b)). Outside the isotropic systems areas, coarse emulsions or gel-like structures were found for both studied surfactant/cosurfactant ratios. MEs were found down to a water concentration of 5% in both cases and up to 75% for the systems containing a higher surfactant level (0.8 ratio) and 65% for the one with lower surfactant level (0.6 ratio). Therefore, the higher level of surfactant did not significantly affect the total area covered by isotropic systems in the pseudoternary diagrams. After this, the study of ME region was carried out again with the formulations containing 4 mM of drug, so as to evaluate if there were significant changes in ME regions. No significant changes in ME regions were observed in both Pseudoternary phase diagram using MEs containing 4 mM of TMX.

This way of research, in which cytotoxicity evaluation is done during the pharmaceutical development process, may result at last, in biological findings more representative; and additionally in a shorter period of time. It is remarkable that Cavalli et al. have recently reported that sometimes the results are partially affected by the conditions of culture medium, as the use of Dimethyl sulfoxide (DMSO) in cytotoxicity assays, for example [27].

### 3.4. Preparation and Solubility Evaluation of Selected MEs Containing TMX

**Figure 6:**
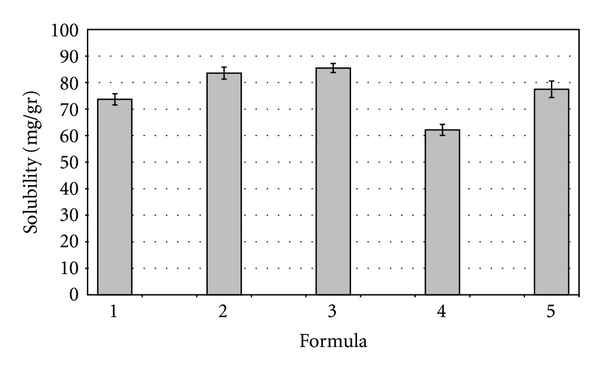
Solubility of Tamoxifen citrate in the selected vehicles. Each bar represents the mean of three samples ± SD (standard deviation for *n* = 3).

Results are shown in Figure 6 and as it can be observed, there is a synergic effect regarding drug solubility in the MEs compared to the solubility in the isolated excipients. This means that, in some cases, the difference observed for solubilizing capacity is tenfold higher.

 Taking into account the composition of the MEs, the solubility seems to increase with the raise in the lipid phase content. Thus, the higher the surfactant percentage for the same lipid level, the higher the solubility in the ME. Considering TMX water solubility (*≈*0.4 *μ*g/mL) [28–30], these systems represent an improvement of around 150000 fold for vehicle 4, which exhibited a solubility of 60 mg/g.

### 3.5. Physicochemical Characterization

A significant lowering effect of approximately 1.5 points in pH values was observed when TMX was added. Conductivity values obtained for the selected compositions correspond to those of o/w MEs [31, 32].

The low viscosity values are representative for MEs (Table 2). The differences observed for viscosity values might be the result of the interaction between ME droplets in oil/water systems. It is expected that PS 80 hydrophilic chains are strongly hydrated and connected with hydrogen bonds; this allows the interaction between the droplets, thus raising the viscosity values [33]. It is also to remark that the higher PC concentrations in the compositions, the higher viscosity was observed.

All selected formulations were nonbirefringent when analyzed with the polarized microscope, confirming their isotropy. It was concluded that MEs were not electrically charged (*z* potential equal to 0 mV) due to their ionic characteristics and dipolar attributes.

**Figure 7:**
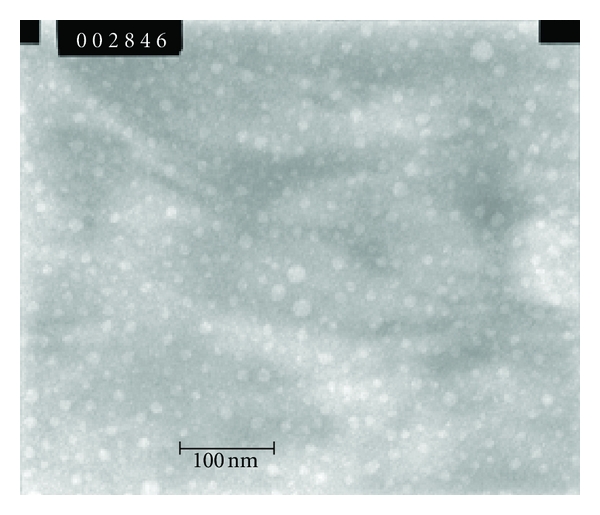
TEM photograph of Formulation 2 (×100000; dilution 1 : 40).

Since ME formation process is generally a random stirring process; the resulting delivery system may result in a polydispersed system in which different droplet sizes can coexist. This information is extremely valuable in practice because both stability and viscosity depend on the drop size distribution [34]. The later in vivo or in vitro behavior depends on this property as well [35]. Results shown in Table 3 are in the typical range for a ME composition with a narrow range of polydispersion as the polydispersity index (PDI) shown [7]. TEM images also confirmed this size distribution (Figure 7) for blank ME N° 2. The addition of TMX did not significantly change droplet size of formulations comparing with empty ones, even at the highest TMX (20 mM). This is an interesting advantage for the selected compositions, because the loading of a lipophilic active compound could result in an increase in the droplet size and, eventually, could compromise the system physical stability [35].

A short stability testing was carried out with selected formulations. For this purpose, TMX 10 mM was loaded in order to achieve a final concentration of approximately 5.10–4 M in the culture media as the higher dose, according to literature data [36]. Results demonstrated that all formulations showed a 100 ± 2% of the initial content after a month of observation. Obtained values confirm the total solubilization of the drug and absence of rapid degradation (data not shown). Regarding physicochemical values, no significant changes in the values measured at the beginning of the study were obtained after the studied period. No precipitation or change in appearance was observed by direct visual observation. None of the fifteen ME formulations has shown any sign of in-stabilization during the thermodynamic stability tests carried out.

### 3.6. In Vitro Performance of Selected MEs

**Figure 8:**
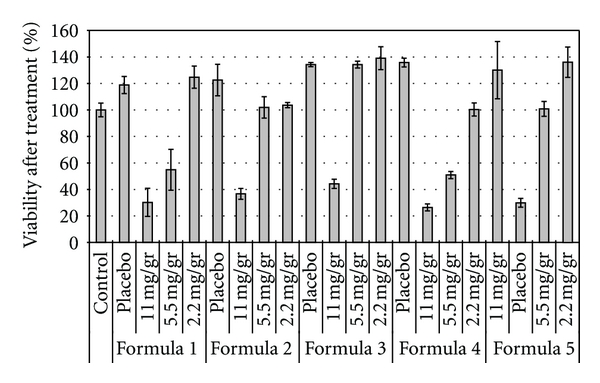
Cell viability of MCF-7 breast cancer cells incubated with empty microemulsions and formulations containing Tamoxifen citrate in the following concentrations: 11 mg/g (20 mM), 5.5 mg/g (10 mM), 2.2 mg/g (4 mM). Each bar represents the mean of three samples ± SD.

As a preliminary experiment, the five empty MEs were cultured to assess if they have any effect on cell proliferation in presence of 10 nM of estradiol. Two controls were also included: one adding estradiol (10 nM) to the cells in order to determine its proliferation effect and the other containing only the cells. As it is shown in Figure 8, none of the empty ME showed effects *per se* over the MCF-7 cell line; it can be observed, instead, the proliferative effect of estradiol on MCF-7 cell line. Results confirmed that the dilution adopted was not cytotoxic.

 The five selected formulations were loaded with the following TMX concentrations, 11 mg/g (20 mM), 5.5 mg/g (10 mM) and 2.2 mg/g (4 mM); it is important to remark that the in vitro performance of selected MEs was carried out in a culture media containing estradiol 10 nM.

The percentage of cellular viability of MCF-7 cells following inoculation of the above-mentioned TMX concentrations is illustrated in Figure 8. There was a significant decrease in cell growth for all formulations containing the highest concentration of TMX. The viable cell percentages after treatment were around 30 to 40% in all cases, that is, at least 90% less of viable cells than the empty compositions; ME N° 4 was the one which shown the highest cytotoxic effect. The same behavior was shown by the formulations 1 and 4 with the intermediate concentration of drug; in these cases the differences shown were 75% and 90%, respectively.

This cytotoxic effect was not observed when formulations N° 1, 3, and 5 were loaded with 4 mM of TMX. But it is to remark that both ME N° 2 and ME N° 4 showed a significant lower number in viable cells when loading this drug concentration. Additionally, it is worth noting that formulas 1, 4, and 5 showed a dose dependent effect. Formulations 2 and 3 did not show significant differences between the effect exerted by 10 mM and the 4 mM TMX concentrations. The TMX suspension was not able to significantly decrease the number of viable cells in any cell culture condition (data not shown).

Even though TMX mechanism of action has not been completely elicited, it was reported that it acts primarily through estrogen receptors (ERs) by modulation of gene expression that finally leads to cell cycle arrest. However, it has been informed that at higher concentrations could induce breast cancer cell apoptosis [36]. This is an ER independent and nongenomic effect; it was found in ER negative breast cancer cells and other cell types such as malignant gliomas, pancreatic carcinomas, and melanomas. On the other hand, estradiol has an antiapoptotic influence in both, ER positive and negative cells, in addition to its proliferative effect on ER positive cells; the antiapoptotic effect has also been reported in MCF-7 breast cancer cell line [37].

From the results obtained in cell cultures, it might conclude that all the compositions containing 20 mM of TMX showed an important cytotoxic effect. This phenomenon would be related with the induction of cellular apoptosis described above; the effect was also observed in ME N° 1 and 4 containing 10 mM TMX.

The % of viable cells observed would indicate that seven of the fifteen assayed compositions were able to solubilize an enough amount of TMX capable to show a modification in the apoptosis cellular induction. It is also interesting to remark that this phenomenon is observed in presence of the above demonstrated proliferative effect of estradiol.

It can be concluded that formulations 1 and 4 had the best in vitro performance because they were able to show an important antiproliferative effect even when they were loading the intermediate dose.

Another interesting observation to point out is that formulation 3 showed the highest percentage of cell viability at any TMX concentration; this formula is the one which has the highest PC (16%) concentration. Previous reports showed that PC content is increased in cancer cells and have an important role in their proliferation [38, 39]. So, it is expected that this stimulation on cell proliferation can be attributed to the levels of PC. This observation and the mechanism described above suggest that the proposed MEs would present a high cellular uptake; anyway, PC proliferation effect has to be considered in further pharmacotherapeutic evaluation.

The obtained MEs are promising in the current state of increasing interest for nanocarriers that can be used for TMX delivery. For example, Chawla and Amiji, examined biodegradable polymeric nanoparticles uptake and distribution in MCF-7 breast cancer cell line. They compared TMX intracellular concentration when delivered by the nanoparticles and in solution, and they found that the drug uptake from the nanoparticles followed a saturable transport. Therefore, above certain concentration, TMX intracellular concentration was much higher when delivered by the solution [1]. On the contrary, MEs designed in this work did not show signs of limited transport in none of the selected drug concentrations. Besides, it is expected that MEs can improve drug cellular uptake not even for a better drug solubilizing capacity but also for the improvement in biopharmaceutical parameters that have been extensively described for them [4, 7, 8, 13, 15, 17, 18].

Al Haj et al. evaluated TMX-loaded solid lipid nanoparticles for parenteral administration, and, though promising, these systems required a sophisticated preparation method because they were elaborated by high pressure homogenization technique [40]. Instead of this, the ease of preparation is a common ME characteristic.

Tagne et al. evaluated a nanoemulsion containing TMX that has a significantly better in vitro performance reducing cell proliferation when compared to a TMX-loaded suspension. However, they have used a concentration of TMX equal to 3 × 10^-5 ^M for all the cell culture treatments, while our MEs were able to solubilize more than 100-fold higher of TMX [6]. These authors claimed for an important cellular uptake because of the nanometric sizes of the nanoemulsions. Similar results could be expected with our formulations but the in vivo therapeutic parameters would be improved because of the drug concentration achieved.

Another important difference between both works is the technique of preparation. They used a microfluidizer processor which provides a resultant high shear rate by accelerating the product through microchannels to a high velocity for size reduction to the nanoscale range. They previously prepared a suspension of TMX and then the mixture was homogenized. On the contrary, MEs involve a spontaneous process of formation for a defined composition and the selection of the composition is searched through a screening of components. As a result of these two different techniques they found a negative *z* potential while we observed no charges on the droplets' layers. Another consequence was that they obtained a bimodal distribution of mean droplet sizes; on the contrary, we observed a more uniform distribution. In conclusion, the above-mentioned differences are in relation with the fact that Tagne et al. have prepared nanoemulsions, while our work deals on MEs; it is very clear in literature the differences between them independently that they could have similar compositions and mean droplet size [4, 8, 41].

More recently, the electrospray technique was proposed to produce TMX-loaded poly(amidoamine)-cholesterol conjugate nanoparticles in powder form without any excipient in a single step. Spite of this, the nanoparticles showed sizes higher than 200 nm and a drug loading of about 40% [27].

It is also necessary to remark that the cell culture experiments were carried out with no reagent addition; this is a very important issue because previous report [27, 42] found that MCF7 cells are highly sensitive towards DMSO. Indeed, volumes equal to or higher than 2 *μ*L (2% v/v) result in a cytotoxic effect that partially overlaps the one observed in cells treated with free TMX diluted in DMSO. Therefore, this “background” cytotoxicity leads to an overestimation of the free TMX activity. On the contrary, every step done in this work during the development of the experimental design was adjusted so as to strictly evaluate the in vitro behavior showed by each one of the selected compositions.

## 4. Conclusion

The present work describes a novel interdisciplinary rational screening for a ME composition, its optimization, and the corresponding in vitro performance evaluation on MCF-7 breast cancer cell line. The development included physicochemical properties evaluation and drug solubility in selected formulations. The experimental design began with the proposal of extensively studied excipients for the screening, after that, the first criterion adopted for excipient selection was based on solubilizing capacity; then cytotoxic was evaluated. The final criterion of selection was the ability to form MEs shown by each one of the excipients.

It is our opinion that this design layout allows a faster optimization of MEs composition. The drug-loading capacity was investigated using TMX, a poorly water soluble antineoplastic drug, as an active compound model. Non-adherence to oral medication is an increasingly recognized concern in the care of cancer patients and considering that every year, hundreds of thousands of women worldwide are recommended to take TMX for 5 years; a different protocol of treatment would be evaluated. Not only other oral administration protocol but also an IM or IV formulation can finally be proposed after the in vivo experiments. In addition, some other ER-negative cancers, which have also shown to be sensitive to TMX may be further evaluated with MEs' containing different pharmacological doses. Thus, a more efficient drug release profile would potentially prevent the development of cancer cell resistance.

Consequently, these MEs result in a promising alternative for further in vivo evaluation. Finally, Peer et al. mentioned that for rapid and effective clinical translation, the nanocarriers should present some characteristics that these ones do exhibit [2]. They are made with biocompatible, well-characterized, and easily functionalized excipients; they are both soluble and colloidal dosage forms under aqueous conditions which are related to increased effectiveness. And they have a low rate of aggregation and a long shelf life. They would also exhibit differential uptake efficiency in the target cells over normal cells because they show passive targeting.

## Figures and Tables

**Table 1:** Composition of the selected microemulsions after the screening of excipients.

Formula	Polysorbate 80 (%)	PC (%)	Capmul MCM L	Propylene glycol (%)	Ethanol (%)	Water (%)
1	20	8			25	47
2	20	12			25	43
3	20	16			25	39
4	15	8			25	52
5	15	12			25	48
6	20		8		25	47
7	20		12		25	43
8	20		16		25	39
9	15		8		25	52
10	15		12		25	48
11	20		8	25		47
12	20		12	25		43
13	20		16	25		39
14	15		8	25		52
15	15		12	25		48

**Table 2:** Physicochemical parameters measured in the selected microemulsions. Data are expressed as mean ± SD (*n* = 3).

Formula	Viscosity (mPa·s) Empty ME	pH (Empty ME)	pH (Loaded ME)	Conductivity (uS/cm) Empty ME	Density (g/mL) Empty ME
1	45.7 ± 1.8	6.11 ± 0.02	4.62 ± 0.02	71.1 ± 0.9	1.00 ± 0.01
2	59.4 ± 4.3	6.09 ± 0.01	4.62 ± 0.02	40.7 ± 1.1	0.98 ± 0.01
3	79.3 ± 7.7	5.96 ± 0.02	4.67 ± 0.01	65.2 ± 1.6	0.99 ± 0.01
4	21.2 ± 2.3	6.15 ± 0.02	4.54 ± 0.01	42.6 ± 0.8	0.99 ± 0.01
5	29.9 ± 2.2	6.00 ± 0.05	4.61 ± 0.02	40.2 ± 1.1	0.97 ± 0.01

**Table 3:** Mean droplet size for selected empty and loaded microemulsions. PdI: polydispersity index.

Formula	Droplet size (nm) Empty ME	pdI	Droplet size (nm) Loaded ME	pdI
1	5.72	0.344	6.04	0.407
2	5.37	0.237	6.04	0.297
3	5.41	0.256	4.97	0.174
4	9.54	0.365	9.62	0.368
5	8.43	0.389	8.33	0.210
